# Feline Host Range of *Canine parvovirus*: Recent Emergence of New Antigenic Types in Cats

**DOI:** 10.3201/eid0804.010228

**Published:** 2002-04

**Authors:** Yasuhiro Ikeda, Kazuya Nakamura, Takayuki Miyazawa, Yukinobu Tohya, Eiji Takahashi, Masami Mochizuki

**Affiliations:** * University College London, London, United Kingdom; † University of Tokyo, Bunkyo-ku, Tokyo, Japan; ‡ Kyoritsu Shoji Corporation, Chiyoda-ku, Tokyo, Japan

**Keywords:** canine parvovirus, feline panleukopenia virus, interspecies transmission

## Abstract

Since the emergence of *Canine parvovirus* (CPV-2) in the late 1970s, CPV-2 has evolved consecutively new antigenic types, CPV-2a and 2b. Although CPV-2 did not have a feline host range, CPV-2a and 2b appear to have gained the ability to replicate in cats. Recent investigations demonstrate the prevalence of CPV-2a and 2b infection in a wide range of cat populations. We illustrate the pathogenic potential of CPV in cats and assesses the risk caused by CPV variants.

Human health and animal welfare continue to be challenged by rapidly evolving pathogens. Although many details about specific host-parasite systems have been reported, our understanding of host range alteration and the evolution of virulence remains rudimentary. We reviewed the evolution of carnivore parvoviruses with particular reference to *Canine parvovirus* (CPV) infection in cats. These parvoviruses’ molecular and phenotypic evolutionary pattern provides an exemplary system to study pathogen-host relationships and the evolution of virulence, both essential factors for understanding newly emerging infectious diseases.

## Emergence of *Mink enteritis virus* and CPV Type 2 (CPV-2)

**Figure 1:**
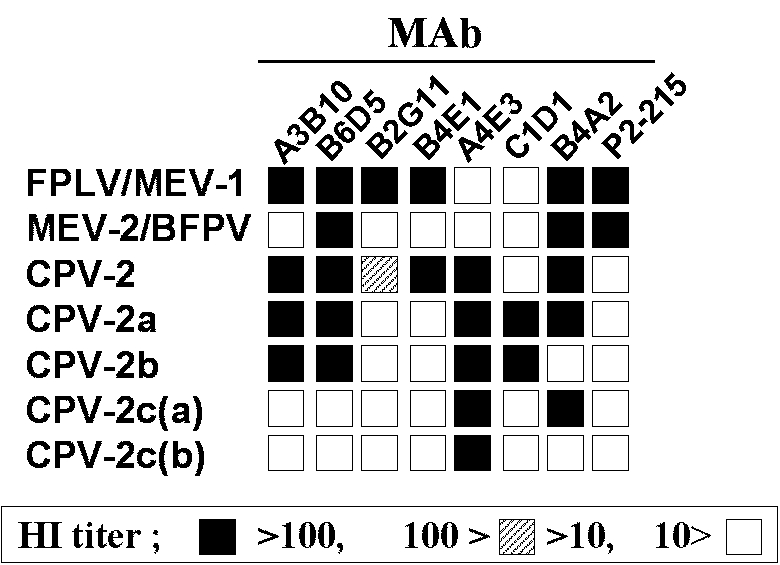
Antigenic profile of feline parvoviruses, including *Canine parvovirus* 2c (CPV-2c) types. Subtype-specific monoclonal antibodies are used to type the viruses in a hemagglutinin-inhibition test (HI). *Mink enteritis virus* (MEV-3) shows similar patterns to MEV-2 (*2*). FPLV = *Feline panleukopenia virus*; BFPV = blue fox parvovirus.

Infection by *Feline parvovirus* was thought only to occur in cats (*Feline panleukopenia virus,* FPLV) or raccoons until the mid-1940s, when a similar disease with a mortality of up to 80% was observed in infected mink kits in Canada (*1*). The disease caused by the mink agent, named *Mink enteritis virus* (MEV), was thereafter observed throughout many regions of the world (*2*). Since MEV was indistinguishable from FPLV by conventional methods such as serum-neutralization assay, MEV isolates have been differentiated from FPLV primarily on the basis of the host from which they are isolated. Using a panel of monoclonal antibodies (MAbs), we now classify FPLV and MEV isolates into three antigenic types, FPLV and MEV type 1 (MEV-1) group, MEV type 2 (MEV-2), and MEV type 3 (*2*,*3*) (Figure 1). MEV-1 and MEV-2 have repeatedly been isolated from the mink in the United States, Europe, Japan, and China (2,3; Y. Ikeda and M. Mochizuki, unpub. data).

**Figure 2:**
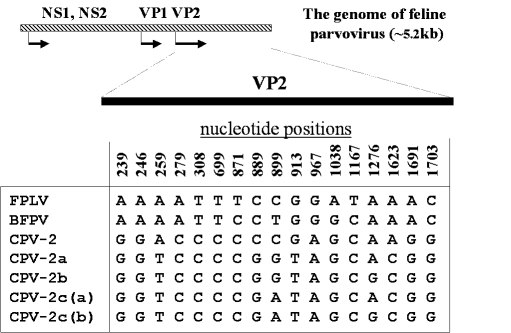
Conserved nucleotide differences between the *Feline panleukopenia virus* (FPLV)- and *Canine parvovirus* (CPV)-type viruses. Nucleotide positions in the VP2 gene are numbered above the sequences; BFPV = blue fox parvovirus.

In the late 1970s, another virus emerged in dogs (*4*,*5*). The new virus, designated CPV-2 to distinguish it from an unrelated *Canine parvovirus* *(Canine minute virus*), spread around the world within a few months (*6*,*7*). CPV-2 spread rapidly, killing thousands of dogs. Polyclonal antibody and in vivo cross-protection studies soon showed that CPV and FPLV were closely related antigenically, while CPV-2 and FPLV were distinguishable from each other when examined with a panel of MAbs (Figure 1). Subsequent extensive genetic analysis of numerous CPV-2, FPLV, and MEV isolates showed that the viruses form two distinct clusters represented by FPLV-type viruses from cats (FPLV), raccoons, and mink (MEV), and by CPV-type viruses from dogs and raccoon dogs. At least 11 conserved nucleotide differences (7 nonsynonymous and 4 synonymous changes) were seen between CPV-2 isolates and FPLV-type viruses in the capsid VP2 sequence; in contrast, CPV and FPLV isolates differ in <2% of their genomic DNA sequences (*8*) (Figure 2; Table).

## Hypotheses on the Ancestor of CPV-2

Retrospective investigations to detect CPV antibodies in sera collected from dogs or related canids showed that the first positive titers were present in European dogs around 1975, while the first positive sera in the USA, Japan, and Australia were seen in early 1978. Various hypotheses on the mechanism of virus evolution in this group have been developed. The most widely accepted hypothesis is the emergence of CPV-2 from a variant of FPLV or of a closely related virus infecting another carnivore, such as mink or foxes (*9*,*10*).

**Figure 3:**
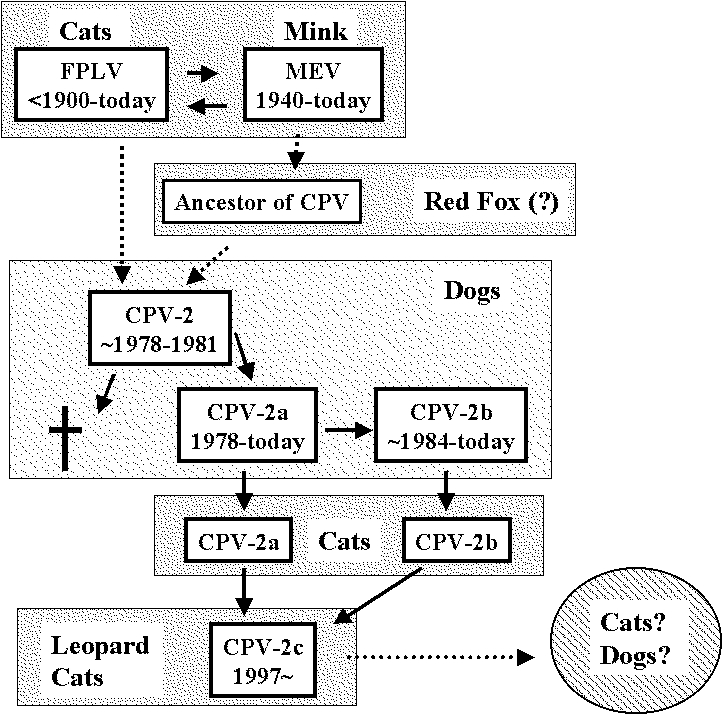
The apparent evolutionary processes of feline parvoviruses.

Several intriguing observations support the latter hypothesis. First, based on the sequence analyses of the capsid VP-2 and the nonstructural NS1 genes, MEV is closer to CPV-2 than FPLV (*9*,*11*). More importantly, the virus isolated from an Arctic fox from Finland (blue fox parvovirus, BFPV) in 1983 appeared to be an intermediate between the FPLV- and CPV-type viruses. BFPV had three synonymous nucleotide changes in the VP2 gene that were specific for the canine sequence (*12*) (Figure 2), while the fox virus was classified antigenically as typical MEV-2-type (*13*) (Figure 1). These findings indicate that some animals in the family *Canidae*, such as mink or foxes, which are susceptible to FPLV-like viruses, might play a role as a reservoir for the ancestor of CPV. Recently, Truyen et al. (*14*) reported that the intermediate parvovirus sequence from a German red fox was CPV-2-like but had one FPLV-specific nonsynonymous substitution. This suggests that German red foxes could harbor the direct ancestor of CPV, although it remains possible that the intermediate red fox parvovirus emerged from conventional CPV-2 by one point natural mutation (Figure 3).

## Emergence of CPV Types 2a and 2b (CPV-2a and CPV-2b)

Since the emergence of CPV-2, two new antigenic types of CPV, designated CPV-2a and CPV-2b, have arisen consecutively. These new virus types have now almost completely replaced CPV-2 viruses as the dominant infectious agents (*15*) (Figure 3). At least four conserved nonsynonymous substitutions have been observed between CPV-2 and CPV-2a isolates in the VP2 gene (Table). CPV-2b isolates have another two nonsynonymous changes from CPV-2a (Table). Although the exact mechanisms of these evolutions are not clear, the emergence of these new antigenic types of CPV can likely be ascribed to the adaptation of CPV-2-type viruses in dogs. Of interest, each new antigenic type has lost at least one neutralizing epitope compared with the former serotype (*16*).

**Table:** Phylogenetically informative amino acid sequences in the VP2 gene

Virus	80	87	93	103	232	297	300	305	323	426	555	564	568
FPLV/ MEV-1	Lys	Met	Lys	Val	Val	Ser	Ala	Asp	Asp	Asn	Val	Asn	Ala
MEV-2/ BFPV	Lys	Met	Lys	Val	Val	Ser	Val	Asp	Asp	Asn	Val	Asn	Ala
CPV-2	Arg	Met	Asn	Ala	Ile	Ser	Ala	Asp	Asn	Asn	Val	Ser	Gly
CPV-2a	Arg	Leu	Asn	Ala	Ile	Ser/Ala	Gly	Tyr	Asn	Asn	Ile	Ser	Gly
CPV-2b	Arg	Leu	Asn	Ala	Ile	Ser/Ala	Gly	Tyr	Asn	Asp	Val	Ser	Gly
CPV-2c(a)	Arg	Leu	Asn	Ala	Ile	Ala	Asp	Tyr	Asn	Asn	Val	Ser	Gly
CPV-2c(b)	Arg	Leu	Asn	Ala	Ile	Ala	Asp	Tyr	Asn	Asp	Val	Ser	Gly

FPLV = *Feline panleukopenia virus*; MEV = *Mink enteritis virus*;. BFPV = blue fox parvovirus; CPV = *Canine parvovirus.*

## Clinical Features of FPLV and CPV in Their Original Hosts

Parvoviruses replicate most efficiently in rapidly dividing cells. Replication is generally lytic, and tissue damage at these sites can be observed (*17*). Infection with FPLV causes two typical syndromes. When infection occurs in fetuses or very young kittens, a distinct cerebellar ataxia is observed when they become actively ambulatory (*18*,*19*). When older kittens are infected, illness characterized by loss of appetite, pyrexia, diarrhea, and leukopenia of both lymphocytes and neutrophils appears (*20*). On the other hand, two typical syndromes observed in CPV-infected dogs are acute myocarditis in young puppies with a high mortality (*21*) and hemorrhagic enteritis in older puppies (*4*,*22*).

Mortality from FPLV infection is likely to depend on the general condition of the animals before infection. Experimental infection of specific pathogen-free (SPF) or germfree cats with FPLV generally leads to mild diseases (*23*,*24*). No or slight intestinal lesions can be observed in infected germfree cats (*23*), which suggests that the intestinal lesions are caused by secondary bacterial, rather than primary viral, infection.

## Host Range of FPLV- and CPV-Type Viruses

The host ranges of FPLV- and CPV-type viruses have been extensively studied in vitro. In general, CPV-type viruses replicate efficiently in feline and canine cell lines, while most FPLV and FPLV-like viruses can replicate efficiently only in feline cells (*11*,*25*–*27*). Subsequent recombination mapping and site-directed mutagenesis experiments have clearly shown that the VP2 gene (including the differences of VP2 residues 93, 103, and 323; Table) is important in controlling canine host range, although a part of the nonstructural NS gene of CPV also participates in FPLV replication in canine cells (*27*,*28*). Recently, Ikeda et al. (*11*) reported a unique FPLV isolate from a domestic cat, which could replicate efficiently in a canine cell line. Interestingly, this isolate was antigenically FPLV-type but had a natural mutation of VP2 residue 323 Asp to CPV-specific Asn, supporting previous site-directed mutagenesis studies. Moreover, FPLV-type virus actually has the potential to acquire canine host range by natural mutation, although phylogenetic analyses indicate that the isolate is unlikely to be a direct ancestor of CPV-2 (*11*).

The in vivo host ranges of FPLV and CPV seem to be more complicated. FPLV can replicate in feline tissues, such as lymph nodes, thymus, spleen, or intestinal epithelial cells, and high titers of virus are shed in feces. In dogs, however, FPLV replication is seen only in the thymus and bone marrow, not in the gut or mesenteric lymph nodes (*26*), resulting in no virus shedding in feces (*29*). In terms of viral evolution, the CPV ancestor had only to gain the ability to infect the gut in order to be shed and spread in the dog population (*26*). Indeed, Mochizuki et al. (*30*) report the isolation of FPLV-type virus from diarrheic feces of a clinically diseased dog. Although the reason why the antigenically FPLV-type virus could gain canine host range remains to be determined, the virus possibly had some genetic mutation(s) that did not change its antigenic properties yet rendered the virus able to infect canine gut cells.

Until recently, the feline host range of CPV has been controversial. Goto et al. report that CPV replicates in cats in a pattern similar to FPLV (*31*); other studies find no detectable CPV replication in any feline tissue (*26*,*32*). This discrepancy, however, is due to the antigenic differences of the examined viruses. The virus (Kushiro strain) used in the first study (*31*) was shown to be CPV-2a (*27*), whereas the other studies used CPV-2 (26,32). Truyen et al. (*33*) directly compared the feline host ranges among CPV-2, CPV-2a and CPV-2b and showed efficient replication of both CPV-2a and CPV-2b in experimentally infected cats. CPV-2a and CPV-2b isolates replicate to high titers in lymphoid and intestinal tissues, while the CPV-2 isolate used in this study did not replicate in experimentally infected animals (*33*).

## Feline Host Range of CPV in the Wild

In late 1980s, CPV was first recognized in cats in a natural setting (*30*). Mochizuki et al. (*30*) examined eight feline isolates collected during 1987 to 1991 in Japan and demonstrated that three were antigenically and genetically identical to CPV-2a viruses. The first isolation of CPV-2a-type virus from a cat was in 1987 (*30*). All three CPV-2a-type viruses were isolated from the feces of clinically healthy cats, while the isolates from cats with typical feline panleukopenia were all conventional FPLV-type. Subsequently, CPV-2a and CPV-2b viruses were recognized in cats in the USA (2 of 20 isolates) and Germany (3 of 36 isolates) (*33*).

Recently, Ikeda et al. (11) examined 18 isolates from unvaccinated cat populations and demonstrated that 15 of the isolates could be classified as CPV-2a- or 2b-related viruses (*11*). Since carnivore parvoviruses are likely to spread freely and rapidly in the environment when few cats and dogs are vaccinated against FPLV or CPV, CPV-2a/2b-type viruses seem to have more advantages over conventional FPLV in cats under natural conditions. It is therefore possible that CPV-2a/2b-type viruses will replace FPLV-type viruses as the dominant infectious agents in domestic cats even in developed countries, where FPLV vaccines are commonly used.

## Emergence of New Antigenic Types of CPV (CPV-2c) in Cats

Feline parvoviruses continue to evolve. CPV-2a and 2b have been detected not only from domestic cats but also from wild felids worldwide (*11*,*34*). Steinel et al. (*34*) report the detection of CPV-2b-type viral DNA from one fecal sample of a Namibian farmed-raised cheetah and the tissue sections of four captive cheetahs in the United States. CPV-2a-type sequence was also found in the fecal sample of the Siberian tiger from a German zoo (*34*).

During 1996 to 1997, CPV-2a/2b-related viruses were isolated from Asian small wildcats, leopard cats *(Felis* *bengalensis*), in Vietnam and Taiwan (*11*,*35*). These viruses were designated as leopard cat parvovirus (LCPV).Three of the six isolates were demonstrated to be new antigenic types of CPV; the other three isolates were essentially identical to CPV-2a or 2b. Subsequently, the new antigenic type viruses were shown to have a natural mutation of VP2 in common (*11*) (residue 300 Gly to an Asp, Table), which results in remarkable changes of their antigenic properties. The new antigenic type, characterized by the loss of the VP2 epitopes recognized by the reference MAbs A3B10, B6D5, and C1D1, is currently designated as CPV-2c (Figure 1) (*11*). The reactivity against MAb B4A2, which distinguishes CPV-2b from the other serotypes, further classifies the CPV-2c-type isolates into two serotypes, CPV-2c(a) and CPV-2c(b) (Figure 1).

CPV-2c-type viruses have been isolated only from leopard cats but not from domestic cats in the same area. Since the phylogenetic analysis indicated that CPV-2a and CPV-2b-type viruses were likely to evolve to CPV-2c(a) and CPV-2c(b)-type viruses, respectively, the mutation at the residue 300 Gly to Asp is probably ascribed to the adaptation of CPV-2a/2b-type viruses to leopard cats. Similar to the emergence of CPV-2a and CPV-2b, CPV-2c has lost neutralizing epitopes compared with the former serotypes, CPV-2a and 2b.

## Virulence of CPV-2a and -2b in Cats

The pathogenicity of CPV-2a and 2b in cats remains debatable. Mochizuki et al. reported the isolation of CPV-2a from a cat manifesting clinical signs of feline panleukopenia (*36*). The detection of CPV-2a/2b-type DNA sequences from the cheetahs with chronic diarrhea and enteritis or the tiger with anorexia and diarrhea (*34*) strongly suggests CPV-2a’s and CPV-2b’s pathogenic potential in large felids. In sharp contrast, recent studies using SPF cats experimentally infected with CPV-2a or CPV-2b showed no or slight illness, such as mild lymphopenia, in the infected animals (*31*,*33*,*37*,*38*). Moreover, the fact that many CPV-2a- and COV-2b-type viruses were isolated from clinically healthy cats (*11*,*30*,*35*,*39*) seems to indicate their relatively low pathogenicity.

At present, this discrepancy remains to be resolved. Note, however, that the experimental infection of SPF cats with FPLV generally leads to mild disease (*23*,*24*). In this regard, the study reported by Goto et al. (*31*) is intriguing. These researchers compared the clinical signs of five SPF and four conventional cats experimentally infected with CPV-2a. The infected five SPF cats showed neither clinical signs nor leukopenia through the observation period, while depression (four cases), vomiting (two), diarrhea (one) , and severe leukopenia (four) were observed in the four conventional cats. One cat died 4 days after infection (*31*). These data indicate that the illness from CPV-2a/2b infection highly depends on the general condition of the cats before infection.

## Pathogenic Potential of CPV-2c

Since feline parvoviruses shed in the feces survive in the environment for up to several months, a fecal-oral route is considered to be the predominant means of their transmission. Although CPV-2c-type viruses have been isolated only from leopard cats (*13*,*38*), the new serotype viruses will likely spread to domestic cat and dog populations. Nakamura et al. (*38*) compared the virulence between FPLV, CPV-2a, and CPV-2c in SPF cats. In this experiment, diverse pathogenicity of the CPV-2a for individual cats was observed. One cat had symptoms frequently associated with parvovirus infection, including leukopenia and diarrhea; the other cats remained asymptomatic. One cat showed no evidence of infection. In contrast to the results obtained with CPV-2a-inoculated animals, all cats inoculated with CPV-2c developed diseases, although the symptoms were relatively milder than those observed in FPLV-inoculated cats. These data indicate that CPV-2c and CPV-2a both have the potential to cause diseases in cats, with some variations of symptoms. CPV-2c appears to be more infectious in cats than CPV-2a and to induce a higher frequency of disease than CPV-2a, although the numbers of cats tested in the experiment were small. Since CPV-2a did not produce any clinical symptoms in the infected SPF cats, yet demonstrated strong virulence in the infected conventional cats (*31*), it is also possible that CPV-2c infection results in severe illness in conventional cats.

The virulence of CPV-2c in dogs remains to be determined. The most probable hypothesis is that the new antigenic viruses can infect dogs and cause some illness, as seen in the emergence of CPV-2a and 2b in 1980s. However, the CPV-2c-type viruses may also have lost their canine host range. The latter hypothesis is based on the fact that CPV-2, which is believed to have emerged from FPLV-related viruses, fails to infect cats. The pathogenic potential of CPV-2c in dogs needs to be addressed (Figure 3).

## Persistent Infection of CPV in Cats

Animals that recover from feline parvovirus infection retain high specific neutralization antibodies and show no virus shedding. Although isolation of FPLV from apparently healthy cats has been reported, feline parvoviruses are generally believed to be completely eliminated from recovered animals.

As mentioned, CPV-type viruses have been isolated from the fecal samples of apparently healthy cats (*30*). Moreover, many CPV-type viruses were isolated from the peripheral blood mononuclear cells (PBMC) of cats with high specific neutralizing antibodies (*11*,*35*,*39*), suggesting that CPV could persistently infect cats irrespective of the presence of the neutralizing antibodies. Although precise mechanisms of the persistent infection of CPV remain to be determined, PBMC probably play a role as a reservoir for the viruses. If one assumes that CPV actually infects cats persistently, examination will be needed to determine whether sporadic shedding of the virus occurs in recovered cats.

## The Efficacy of Conventional FPLV Vaccines against CPV

The study of an attenuated live FPLV vaccine for CPV-2b infection has shown that vaccinated SPF cats are protected from challenge with CPV-2b at 2 weeks after vaccination (*37*). A cross-neutralization study of the antibodies induced by an inactivated FPLV vaccine demonstrated that the vaccinated cats actually develop neutralizing antibodies against CPV-2a, 2b, and 2c as well as FPLV (*40*). These data indicate that commercially available FPLV vaccines can be used for protection against CPVs, at least in the short term. However, antibody titers induced by a FPLV vaccine are significantly lower against CPVs than FPLV (*40*). Indeed, CPV infection was observed in the cheetahs vaccinated with a killed FPLV vaccine (*34*). We therefore suggest that FPLV vaccines are not always sufficient to protect cats from CPV infection in the long term. Steinel et al. (*34*) have proposed the need for inactivated vaccines that use CPV-2a or 2b for cats. CPV-2a/2b-based vaccines are expected to protect cats more efficiently from CPV infection than conventional FPLV vaccines. Recently, Nakamura et al. reported that cats experimentally infected with CPV-2a develop high titers of neutralizing antibodies against CPV-2a and 2b but show relatively low titers against FPLV (*40*). Thus, like FPLV vaccines for CPV infection, CPV-2a/2b-based vaccines may be less efficient for FPLV infection, which would be a major concern. Interestingly, CPV-2c-infected cats showed similar neutralization antibody titers against FPLV, CPV-2a, and 2b as well as CPV-2c (*40*). An inactivated CPV-2c-based vaccine for cats could be a promising vaccine candidate against both CPV and FPLV infection.

## Problems with the Current Parvovirus Nomenclature

Finally, we point out problems with the current nomenclature of carnivore paroviruses, including FPLV, MEV, and CPV. As we mentioned, all carnivore parvovirus isolates are known to be genetically closely related to each other; interspecies transmissions readily occur among carnivores. On the other hand, field isolates have been distinguished on the basis of the host from which they are isolated. According to this system, CPV-2a- and 2b-type isolates from cats should be designated as FPLV types 2 and 3, even though they are essentially indistinguishable from CPVs from dogs. To solve this problem, a new nomenclature is needed. Naming any field carnivore isolate as feline parvovirus or carnivore parvovirus, irrespective of their original hosts, and using the names such as FPLV and CPV-2a to distinguish antigenic or genetic properties would be more appropriate.

## Conclusion

The evolutionary pattern of FPLV in cats differs from that of CPV in dogs. Since FPLV is in evolutionary stasis in cats, FPLV mainly evolves with random genetic drift (*9*). In contrast, CPV appears to evolve in dogs under certain positive selection on the VP2 protein (*9*), which may be because of its short history in dogs. How CPVs are evolving in cats remains relatively obscure. Since CPV-2a and 2b are likely to act as newly emerging parasites in cats, some cat-specific positive selection(s), such as relative in vivo fitness and immune surveillance, could operate as a driving force of CPV evolution. The emergence of CPV-2c in leopard cats is a good example of the evolution of CPV in new hosts. Similarly, since specific antibodies against CPV have been detected in a wide range of wild animals, such as large felids, wildcats, civets, otters, and even bears, such interspecies transmissions probably result in accelerated emergence of other new antigenic types of CPVs because of the new host-specific positive selection.

Elucidating how feline parvoviruses are evolving and how newly emerging variants behave may help to prevent a possible outbreak of the new variant. Assuming that a new virulent CPV variant emerges in cats in the future, what can we expect? Fortunately, the newly emerging variant will not likely cause rapid outbreaks in cats or dogs, since FPLV and CPV-2a/2b have been actively circulating in carnivore populations. Commercially available FPLV or CPV-2-based vaccines might also protect animals from the new virus infection. However, if the new virus gains wider host ranges, deadly outbreaks could be observed, as when CPV-2 emerged in dogs. In any case, recent isolates need to be investigated to anticipate and assess the risk caused by newly emerging viruses.
